# Cue reactivity and its relation to craving and relapse in alcohol dependence: a combined laboratory and field study

**DOI:** 10.1007/s00213-015-4027-6

**Published:** 2015-08-11

**Authors:** Jurriaan Witteman, Hans Post, Mika Tarvainen, Avalon de Bruijn, Elizabeth De Sousa Fernandes Perna, Johannes G. Ramaekers, Reinout W. Wiers

**Affiliations:** Faculty of Humanities, Leiden University Center for Linguistics, Leiden Institute for Brain and Cognition, Leiden University, Leiden, The Netherlands; VICTAS Addiction centre, Utrecht, The Netherlands; University of Eastern Finland, Kuopio, Finland; Dutch Institute for Alcohol Policy, Utrecht, The Netherlands; Maastricht University, Maastricht, The Netherlands; University of Amsterdam, Amsterdam, The Netherlands

**Keywords:** Addiction, Alcohol, Cue reactivity, Relapse

## Abstract

The present study investigated the nature of physiological cue reactivity and craving in response to alcohol cues among alcohol-dependent patients (*N* = 80) who were enrolled in detoxification treatment. Further, the predictive value with regard to future drinking of both the magnitude of the physiological and craving response to alcohol cues while in treatment and the degree of alcohol-cue exposure in patients’ natural environment was assessed. Physiological reactivity and craving in response to experimental exposure to alcohol and soft drink advertisements were measured during detoxification treatment using heart rate variability and subjective rating of craving. Following discharge, patients monitored exposure to alcohol advertisements for five consecutive weeks with a diary and were followed up with an assessment of relapse at 5 weeks and 3 months post-discharge. The results indicated that the presence of alcohol cues such as the portrayal of the drug and drinking behaviour induced physiological cue reactivity and craving. Additionally, cue reactivity and craving were positively correlated, and cue reactivity was larger for patients with shorter histories of alcohol dependence. Further, patients reported a substantial daily exposure to alcohol cues. The magnitude of cue reactivity and the craving response to alcohol cues at baseline and degree of exposure to alcohol cues in patients’ natural environment did not predict relapse. It is concluded that the presence of alcohol cues such as portrayal of alcoholic beverages and drinking behaviour induces cue reactivity and craving in alcohol dependence through a conditioned appetitive response.

## Introduction

Alcohol dependence can be regarded as a chronic condition (Koob and Volkow 2009), characterized by high rates of relapse into problematic drinking soon after initial successful treatment (Witkiewitz and Marlat 2007). Insight into what factors promote relapse could provide a starting point for developing treatments that reduce relapse. The present study aimed to test the influence of one such factor, exposure to alcohol-related cues, by measuring the physiological and craving response to alcohol cues in the laboratory and the influence of naturally occurring alcohol cues (i.e., alcohol advertisement) in the daily environment of alcohol-dependent patients on relapse.

A large evidence base suggests that an exaggerated central nervous system response to alcohol-related cues is a key phenomenon in alcohol dependence (Bechara 2005; Koob and Volkow 2009; Wiers et al. 2007). A prominent hypothesis about the mechanism behind reward circuitry hyperactivity for alcohol-related cues in alcohol dependence is the ‘incentive salience’ hypothesis (Robinson and Berridge 2008), which proposes that initially neutral cues (such as the sight of a beer bottle) by repeated pairing with the direct pharmacological effect of alcohol on the reward circuitry gain ‘incentive salience’ through classical conditioning and can ultimately also evoke a response of the reward circuitry in the absence of the direct pharmacological effect. The magnitude of this conditioned reward circuitry response has subsequently been hypothesized to be associated with craving (Lit and Cooney 1999; Volkow et al. 2012), ultimately promoting relapse (Niaura et al. 1988; Volkow et al. 2010).

Indeed, recent meta-analyses of the neuroimaging literature of alcohol cue exposure have indicated that in alcohol dependence, there is hyperactivity of the reward system for alcohol-related cues as compared to controls (Kühn 2011; Schacht et al. 2013). Further, studies have confirmed a relationship between the magnitude of physiological reactivity to alcohol cues during treatment and subsequent probability of relapse after discharge (Rohsenow et al. 1994; Grüsser et al. 2004; Beck et al. 2012; Garland et al. 2012; but see Heinz et al. 2007). For baseline cue-elicited craving during treatment however, results have been mixed with two studies showing a relationship between the magnitude of the craving response during treatment and subsequent relapse (Cooney et al. 1997; Papachristou et al. 2014) while another study (Rohsenow et al. 1994) did not find such a relationship. Interestingly, Rohsenow et al. (1994) measured both craving and physiological (salivation) response to alcohol cues, allowing for a direct comparison of predictive validity and found that physiological cue reactivity did but craving did not predict future drinking behaviour.

This apparent differential predictive value of physiological responses to alcohol-related cues on the one hand and the craving response on the other hand suggests that these two phenomena may be caused by partially overlapping (but also distinct) mechanisms. Indeed, previous studies have shown that physiological cue reactivity does not (Erblich et al. 2011) or does only moderately correlate (Myrick et al. 2003; Wrase et al. 2007; Mason et al. 2008; for discussion see Carter and Tiffany 1999) with subjective craving, which would be expected if these two phenomena were only partially overlapping. One explanation for this moderate relationship is that craving reflects cue reactivity of the reward system but also additional processes, such as the (verbal) interpretation of the physiological response (Rohsenow et al. 1994; Carter and Tiffany 1999; Drummond 2000). Subjective craving is likely to be influenced by factors such as demand characteristics, resulting in moderate correlations between cue reactivity and craving. Elaborative interpretation of cue reactivity might additionally explain variance in relapse rates, as suggested by the ‘Elaborated Intrusion Theory’ (May et al. 2015). Additionally, factors such as variation in interoception (Verdejo-Garcia et al. 2012) and cognitive control (Volkow et al. 2010) might mediate the relationship between physiological reactivity to drug cues and craving. Thus, in the present study, both physiological cue reactivity and cue-elicited craving to alcohol cues were measured in order to test the purported differential predictive power of these two phenomena with regard to future drinking behaviour.

An abundant and salient source of alcohol cues in society is an alcohol advertisement. Thus, an alcohol advertisement may act as a conditioned stimulus and engage the sensitized reward system (Tapert et al. 2003) and subsequently induce craving and motivation to drink among alcohol-dependent patients. Indeed, one study showed hyperactivity of the prefrontal cortex and thalamus and higher craving for alcohol-dependent patients versus controls after exposure to a printed alcohol advertisement (George et al. 2001). Similarly, alcohol-dependent adolescents have been found to show hyperactivity in (inter alia) the reward circuitry after exposure to printed alcohol advertisements as compared to controls (Tapert et al. 2003). Therefore, in the present study, both physiological reactivity and craving in response to an alcohol advertisement was tested when patients were still in treatment. Furthermore, it was assessed whether these factors show a relationship with subsequent drinking behaviour.

What cues in alcohol advertisement, then, might engage the reward circuitry and induce craving and motivation to drink? Staiger and White (1991) suggested that particularly the sight and smell of an alcohol-dependent patient’s favourite drink induces cue reactivity. Thus, this study suggests that cue reactivity due to alcohol-advertisement exposure may be specific to the favourite brand of alcohol-dependent patients. A study by Mucha et al. (2000) using the startle response, however, suggests that specifically observing the preparation of drug use (i.e., people preparing to drink and actual drinking behaviour in the case of alcohol) may be particularly potent in evoking a response. However, both of these studies did not use alcohol advertisements as alcohol cues. Thus, in the present study, it was tested whether observing drug-related cues such as the preparation and actual use of the drug in alcohol advertisement may be particularly potent in eliciting cue reactivity among alcohol-dependent patients.

The conditioned reward circuitry response to drug-related cues is accompanied by activity of the autonomous nervous system (Bechara 2005). Parasympathetic nervous system activity in response to external stimulation can be measured using the High-frequency (HF) heart rate variability (HRV) component (Thayer and Lane 2000). However, only two previous studies have investigated HF HRV responses to drug cues. Erblich et al. (2011) found an increase in HF HRV when smokers imagined a smoking script versus a control script. Similarly, Garland (2011) found an increase in the HF HRV component during stress-primed alcohol-cue exposure among alcohol-dependent patients. However, a smaller study with methamphetamine users failed to find an effect of drug cues on HF HRV (Culbertson et al. 2010). It has been suggested that this HF HRV increase either reflects a homeostatic response to an aversive stimulus (Erblich et al. 2011) or the regulation of an appetitive response to drug cues (Garland 2011). Only one recent study has examined whether the HF HRV response to stress-primed alcohol cues also shows a relation with subsequent drinking behaviour. A larger HF HRV response to stress-primed alcohol cues was associated with an increased probability of relapse (Garland et al. 2012). Thus, HF HRV might be a cost effective and valid psychophysiological marker of relapse vulnerability.

Not only the *magnitude* of the physiological and craving response to alcohol cues while in treatment (Niaura et al. 1988), but also the *degree* of actual alcohol cue exposure in daily life could contribute to the probability of relapse in alcohol dependence. Thus, there is a need to extend lab measurements of cue-elicited craving and physiological cue reactivity with more ecologically valid measures (Lit and Cooney 1999). However, in the previous literature, so far, only lab measurement of craving and cue reactivity magnitude has been examined while the degree of alcohol cue exposure in the natural environment of patients remains unexplored. Therefore, the present study attempted to extend previous work by not only measuring the HF HRV and craving response to alcohol cues experimentally while patients were still in treatment, but additionally the degree of (self-reported) exposure to alcohol cues (more specifically, alcohol advertisement) in the patients’ daily life. It was expected that exposure to alcohol cues would induce physiological cue reactivity and craving. Further, it was predicted that the magnitude of the cue reactivity and craving response to alcohol cues or the degree of actual alcohol cue exposure in daily life, or both, would predict future drinking behaviour.

## Methods

### Participants

A total of 80 alcohol-dependent inpatients who were enrolled into detoxification treatment at Victas addiction centre (Utrecht, The Netherlands) participated in the study. Patient demographic and clinical characteristics can be found in Table 1. The detoxification program was typically followed by an ambulatory cognitive behavioural therapy program. Cue exposure therapy was not part of the detoxification program.

**Table 1:** Patient characteristics (*N* = 79)

Variable	
Age in years, M (SD)	46.3 (10.8)
Male gender, %	70
Admission-baseline test interval in days, M (SD)	9.3 (4.3)
Baseline test-discharge interval in days, M (SD)	4.6 (2.5)
Duration of detoxification treatment in days, M (SD)	14.1 (4.3)
No. of DSM-IV alcohol-dependence symptoms, M (SD)	6.1 (0.9)
No. of standard units of alcohol/day in previous year, M (SD)	13.1 (7.4)
AUDIT score, M (SD)	25.5 (4.7)
Duration of problematic alcohol use in years, M (SD)	13.9 (9.9)
Polysubstance users, %	21.3
Cannabis use in previous year, %	32.9
Cocaine use in previous year, %	18.1
Tobacco use in previous year, %	84.7
MDMA use in previous year, %	2.6
Hallucinogen use in previous year, %	1.3
Stimulant use in previous year, %	6.4
Tranquillizer use in previous year, %	8.9
Opiate use in previous year, %	1.3
Volatile organic compound (VOC) use in previous year, %	5.1
Other substance use in previous year, %	2.5
Psychopharmacologically active agent prescribed, %	25.0

Inclusion criterion were (1) a Diagnostic and Statistical Manual of Mental Disorders – fourth edition (DSM-IV) diagnosis of alcohol dependence for the 12 months leading up to admission to the addiction centre based on the M.I.N.I.-plus International Neuropsychiatric Interview (Sheehan et al. 1998; Van Vliet and De Beurs 2007) and (2) between 18 and 70 years old and (3) currently stable condition as indicated by the cessation benzodiazepine administration as prescribed by the addiction physician for the treatment of withdrawal. Exclusion criteria were (1) the presence of a severe psychiatric (severe depression, psychotic disorder), neurological (e.g., severe amnesia or tremor) or other somatic disease or (2) very low intelligence (based on clinical impression), as these factors would significantly complicate adherence to the study procedures. Alcohol abuse was required to be the main substance-use problem but other substance use than alcohol did not serve as an exclusion criterion in order to increase ecological validity

For a small subset (*N* = 8) of patients, it was decided that they would be enrolled in a longer additional treatment program after participation in the baseline session. These participants were excluded from the longitudinal part of the study (as including them would induce variation in the interval between testing and discharge) but retained for the analyses of baseline measures. Additionally, one patient was excluded because severe neuropsychiatric disorder was suspected based on baseline testing, leaving us with 79 patients for the baseline measurements. For the longitudinal part of the study, 68 patients were enrolled, of whom 65 agreed to additionally participate in the diary part of the study. As can be observed from the number of DSM-IV symptoms endorsed and reported mean number of standard units of alcohol consumed in the previous year in Table 1, alcohol dependence was relatively severe in the current sample, and a significant proportion of patients reported polysubstance use (defined as the use of two or more recreational drugs in addition to alcohol and tobacco at least twice in the previous year). All patients were abstinent from psychoactive recreational drugs during detoxification as measured with routine urinalysis.

### Study design and procedure

All patients who seemed to fulfill the inclusion criteria based on an initial screening were invited to participate in a 1-h baseline session. During this session, it was first verified whether the patient fulfilled the inclusion criteria, and if so, informed consent was obtained. Subsequently, patients performed two tasks, the results of which will be reported elsewhere. In between the two tasks, retrospective measures of drinking quantity (and other drug use) were administered (see Measures section for more details) and clinical background variables assessed. Further, all participants watched a 5-min series of alcohol and a 5-min series of soda advertisements (order of soda versus alcohol advertisement counterbalanced between participants) while HR data was recorded. Patients were instructed to attentively watch the commercials. After each film, patients indicated their current level of craving for alcohol on a VAS scale. Lastly, patients eligible for the longitudinal part of the study (i.e., all patients in the short detoxification program) were asked to participate. For patients enrolled in the longitudinal part of the study, a diary was handed out at the end of the baseline session to monitor alcohol-advertisement exposure following discharge. Relapse was assessed 5 weeks and 3 months post-discharge by a telephone interview that lasted 5 min. The research protocol was evaluated by the ethics committee of Maastricht University, and the study was conducted in accordance with the Declaration of Helsinki. Patients received a 10 € reward voucher for participation in the baseline session which took 1 h to complete and an additional 50 € worth of vouchers for 5 weeks of diary monitoring.

### Measures

#### Substance use and medication

Several indices of the quantity and nature of substance use were obtained. Mean alcohol use in the 12 months before admission was assessed using the Quick Drinking Screen (QDS), a ‘quantity-frequency’ method for assessing alcohol use that correlates highly with the Time Line Followback Method (Sobell et al. 2003). The Alcohol Use Disorders Identification Test (AUDIT) was used to assess the severity of alcohol abuse in the last 12 months (Saunders et al. 1993). Similarly to Joos et al. (2012), age of problematic drinking onset was assessed by asking ‘at what age did you start drinking problematically, according to yourself and/or your environment?’ and used to calculate the duration of problematic drinking. Additionally, frequency of other substance use other than alcohol in the 12 months preceding admission was assessed. Finally, it was assessed whether relapse prevention medication (naltrexone, disulfiram, acamprosate) had been prescribed by the addiction physician and whether the patient used a psychopharmacologically active agent (antidepressant, antipsychotic, stimulant or anticonvulsant).

#### Cue-elicited heart rate variability (HRV) and craving

All participants watched two 5-min films, containing a series of eight soft drink or alcohol commercials. The commercials were pre-existing commercials from brands not available in the Netherlands in order to remove preferred brand-specific effects. The alcohol-marketing collage contained five beer commercials, one wine commercial and two liquor (shooters and vodka) commercials and contained images of beer, wine and liquor, footage of the beverage being poured into a glass and of people consuming these beverages. The soft drink commercials were matched in content to the alcohol commercials.

Cue-elicited HRV was measured using a Polar RS800 CX (Polar Electro Oy, Kempele, Finland) heart rate monitor (HRM) at 1000 Hz. The device collects HR data through a two-lead chest band which wirelessly transmits the data to a wristwatch. Although there has been some discussion concerning the validity of Polar HRM in measuring HRV (Wallén et al. 2012; Quintana et al. 2013), in subjects without heart disease, the measures obtained with Polar HRM in the time domain and for normalised power in the frequency domain show high correspondence with the gold standard, traditional electrocardiography (Weippert et al. 2010).

Cue-elicited subjective craving for alcohol was assessed by asking patients to indicate their current craving for alcohol on a 100-mm visual analogue scale (VAS). Although the use of multi-item instruments to assess craving has gained popularity, it has been shown that VAS scales are reliable in assessing craving (Kozlowski et al. 1996; Papachristou et al. 2013). In the present investigation, it was decided to use a VAS scale because we were interested in acute changes in craving level in response to alcohol cues, which demanded a rapid assessment procedure.

#### Advertisement diary

Patients used prospective diaries to estimate actual exposure to alcohol marketing following their discharge from the clinic. Among self-report measures available, retrospective reports and retrospective diaries suffer from recall bias effects more than a prospective diary (Patrick and Lee 2010). Recall effects can be expected to be prominent in the case of alcohol-advertisement exposure monitoring, as the relevant events are relatively frequent and brief. Therefore, prospective monitoring of an alcohol advertisement using a diary was employed. Using the diary, patients monitored an alcohol advertisement by briefly marking in a table every time an advert was noticed. All relevant advertisement channels (television, film, outdoor advertisement, radio, in shop advertisement) were covered. All patients monitored an advertisement for two days a week, one weekend day and a weekday (particular weekday and weekend day counterbalanced across patients) during 5 weeks following discharge. As a control (and to reduce attention for alcohol cues), patients monitored a soft drink advertisement as well.

#### Assessment of relapse

There is no consensus on a definition of the term relapse (Witkiewitz and Marlat 2007). In the interest of comparability, various measures of relapse were therefore collected through telephone interviews. First, it was assessed whether the patient had consumed any alcohol at all (abstinence), and if so, how many days after discharge the first alcoholic drink had been consumed (‘time to first drink’). Further, the number of days since discharge on which the patient had consumed any alcohol was indexed (‘number of drinking days’). Similarly, we asked every patient whether six or more standard units had been consumed on any occasion (i.e., binge drinking) and if so, what the time to first binge drink was and how many binge-drinking days had occurred. Lastly, we asked patients whether they evaluated the current drinking behaviour as problematic, and if so, whether they evaluated the current problem-drinking behaviour as less severe, equally severe or more severe than pre-detoxification.

### Data analysis

#### HRV pre-processing

Concerning cue-elicited HRV, the raw RR data for each experimental condition were extracted from the HRM and visually inspected for abnormalities. The data were then imported into Kubios HRV software (version 2.0, 2008, Biosignal Analysis and Medical Imaging Group, University of Kupio, Finland, MATLAB). For each series of commercials (alcohol, soft drink) an epoch of exactly 5 min was first automatically checked for artefacts, using the medium artefact correction setting. After a Fast Fourier Transform (FFT), normalised power of the high-frequency (HF) component (0.04–0.15 Hz) was extracted and used as the HRV measure from the frequency domain. It has been shown that the HF component of HRV at least partially reflects parasympathetic control over the heart, particularly with a within-subject analysis as in the present study (Bernston et al. 1997). Patients who reported heart disease (*N* = 3) or who showed abnormalities upon visual inspection of the RR data (*N* = 2) were excluded from the statistical analyses.

In the 5-min series of alcohol advertisements, alcohol cues were only sporadically presented. Therefore, we were interested in whether the presentation of alcohol cues (i.e., the presentation of objects associated with drinking such as a bottle, the pouring of an alcoholic beverage in a glass and people displaying drinking behaviour) in an alcohol advertisement might specifically elicit an HRV response, as predicted by cue reactivity theory. To test this hypothesis, an ‘event-related heart variability’ (EVHRV) analysis was additionally performed as used previously (Slater et al. 2006), for which absolute HF power was extracted from five segments with a duration of 8 s during which alcohol cues were presented (including people consuming an alcoholic beverage) and compared to the immediately preceding 8-s interval that did not contain any alcohol cues (pre-cue baseline). Since the high-frequency component of HRV has a relatively short oscillation time, 8-s segments are in principle sufficient to estimate HF HRV power (i.e., on average, two oscillations per segment can be measured). Because we additionally averaged our estimate of EVHRV HF power over the five 8-s segments (i.e., for a total measurement window of 40 s), estimation accuracy is further improved.

Due to the practical limitation of the requirement that all alcohol-cue segments had to be preceded by an alcohol-cue-free baseline, all alcohol-cue segments were extracted from beer commercials. Similarly, due to practical limitations, for two segments containing soft drink cues, a baseline had to be chosen that occurred after the occurrence of the segment containing soft drink cues.

#### Diary data pre-processing

Total exposure to an alcohol and soft drink advertisement was computed as the sum of all individual alcohol and soft drink exposures over the 5-week interval (comprising a total of ten monitoring days). The amount of exposure prior to relapse was additionally computed as the mean number of alcohol/soft drink ads reported in the period prior to relapse. Lastly, weekly total exposure to alcohol/soft drink ads was computed as the sum of all alcohol/soft drink ads for each of the five monitoring weeks.

#### Statistical analysis

Because both craving VAS-scores and HRV-HF power (except normalised HF for soft drink advertisement) were not normally distributed (for all, Shapiro-Wilk < .9, *p* < .001), Wilcoxon Signed-ranks tests were used to test for the effect of alcohol advertisements on craving and mean HF HRV power (as compared to soft drink advertisement) and the presence of alcohol cues (as compared to the immediately preceding alcohol-cue-free baseline) on EVHRV HF power. However, to investigate whether there was an alcohol-advertisement-specific increase/decrease in HF power as compared to baseline, we additionally performed a repeated-measures ANOVA. The relationship between craving and cue reactivity was examined using non-parametric (Spearman) correlation coefficients.

Hierarchical regression analyses were performed to predict relapse at 5 weeks and 3 months post-discharge, with addiction severity (AUDIT) and addiction duration (number of problem-drinking years), cue reactivity (HRV) and cue-elicited craving at baseline, and self-reported exposure to alcohol-advertisement post-discharge as predictors. More specifically, for all regression analyses, baseline difference scores between the alcohol and soft drink commercials for craving and in the case of HRV the difference in EVHRV HF spectral power between exposure to alcohol cues as compared to baseline (Llabre et al. 1991; Garland 2011) also known as ‘delta’ (∆), were used as predictors. Similarly to Garland (2011), these three classes of variables (addiction severity and duration; baseline measures; field exposure to advertisement) were entered into the regression analyses in three blocks.

For binary relapse variables (abstinence and binge drinking), logistic multiple regression analyses were performed with mean self-reported exposure to alcohol ads and soft drink ads (as a control) per day *before* the day of relapse (i.e., drinking or binge drinking) as predictor (since exposure after relapse cannot have contributed to relapse). For continuous relapse variables (number of drinking days and number of binge-drinking days), self-reported exposure to alcohol and soft drink ads over the whole 5-week monitoring period was used as the measure of field exposure, as in this case both alcohol-advertisement exposure before and after the first (binge) drink day could theoretically have contributed to the number of drinking days. Because number of (binge) drinking days was not normally distributed (for all, Shapiro-Wilk < .9, *p* < 0.001), these variables were first natural log transformed. Lastly, Cox-regression with the baseline measures as predictors and weekly exposure to alcohol advertisement as time-varying covariates was performed to predict time to first (binge) drink.

We were additionally interested in whether there might be an interaction between baseline measures of responsiveness to an alcohol advertisement (i.e., EVHRV HF power reactivity and craving) and the degree of exposure to alcohol advertisement as measured with the alcohol diary. Therefore, for all dependent variables mentioned above, we ran interaction analyses separately for craving and EVHRV HF power reactivity. We first entered all predictors separately (i.e., either EVHRV HF power or craving and alcohol-advertisement exposure as measured with the diary), and subsequently, the interaction between the baseline measurement and self-reported alcohol-advertisement exposure. In the case of continuous dependents (e.g., number of drinking days), we first demeaned the predictors to avoid co-linearity.

To deal with missing data, multiple imputation was performed (Hallgren and Witkiewitz 2013). The imputation model included all the variables used in the regression analyses. As recommended (Collins et al. 2001), the following auxiliary variables known to be associated with the dependent variable were additionally included: age, gender, number of alcohol-dependence symptoms, total standard units of alcohol consumed in the previous year. We report statistics over the pooled estimates of five imputations, as implemented in the Multiple Imputation procedure in SPSS.

## Results

### Baseline session

**Fig. 1:**
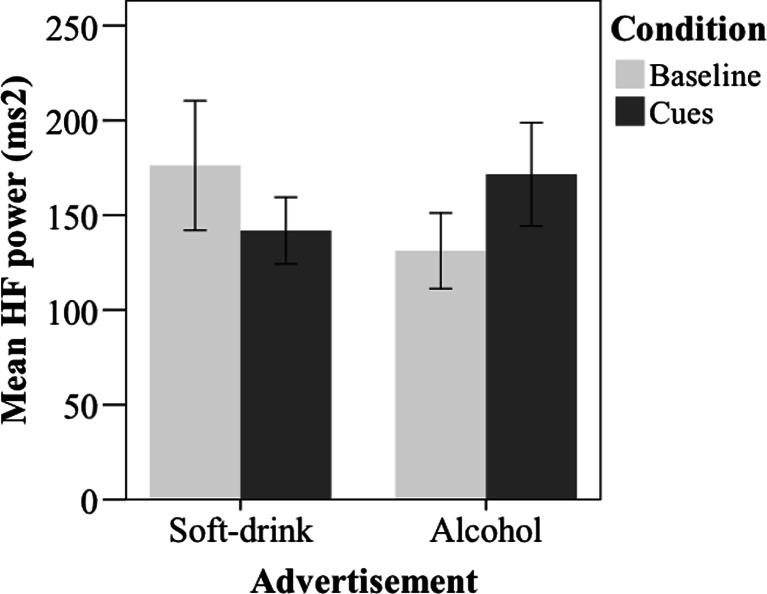
Mean EVHRV HF power during cue exposure and baseline in the block of alcohol and soft drink advertisement. *Error bars* indicate the 95 % confidence interval

HRV measurements for alcohol and soft drink advertisement were available for 67 patients. Wilcoxon Signed-ranks test revealed no significant difference in normalised mean HF HRV power between exposure to alcohol versus soft drink advertisement (*Z* = −1.56, N.S) blocks. HF absolute power measurements during exposure to an alcohol advertisement were available for 71 patients. The test-retest reliability for the mean EVHRV HF power across the eight segments between both baseline measurements was .70, indicating acceptable reliability. For the EVHRV HF power analysis, we first performed a RM-ANOVA with ads (alcohol versus soft drink) and condition (baseline, cue exposure) as the two within-subject factors and, counterbalancing order (soft drinks ads presented first versus alcohol ads presented first) as the between subject variable and HF spectral power for the five combined 8-s segments as the dependent, which revealed the critical significant ads × condition interaction (*F*(1,65) = 13.41, *p =* 0.001) and an ads × order interaction (*F*(1,65) = 8.92, *p* = 0.004). There were no other main effects or interactions. Regarding the order effect, HF power was higher during the alcohol block than the soda block when alcohol ads were presented first, while EVHRV HF power was higher during soft drink than alcohol ad exposure when soft drink ads were presented first. Follow-up Wilcoxon Signed-ranks test for the critical ads × condition interaction revealed a highly significant increase in HF power during the presentation of alcohol cues (*Mdn* = 100.54) as compared to during (*Mdn* = 89.05) pre-cue baseline (*Z* = 2.74, *p* = 0.006). Although soft drink cues decreased HF power as compared to baseline, this decrease was not significant (see Fig. 1). Using Thayer et al. (2002), we estimated the respiratory frequency during the EVHRV segments to be in between 0.23 and 0.24 Hz; hence, respiration could not have significantly affected our HF power measurements.

**Fig. 2:**
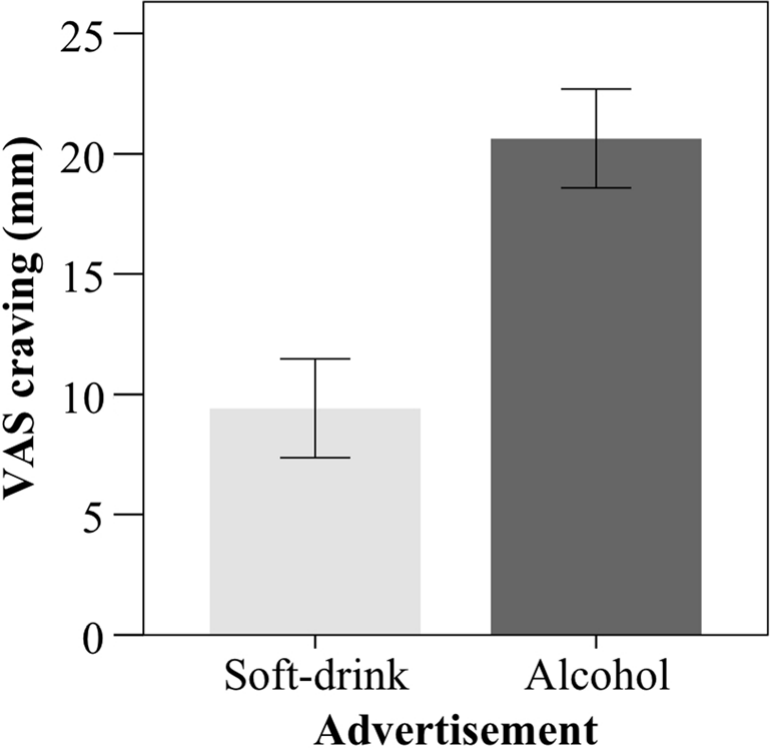
Mean craving score after alcohol and soft drink advertisement exposure. *Error bars* indicate the 95 % confidence interval

Craving VAS-scores after soft drink and alcohol-advertisement exposure were available for 79 patients. Wilcoxon Signed-ranks test indicated a significantly higher craving level after exposure to an alcohol (*Mdn* = 14) advertisement as compared to the level of craving after a soft drink (*Mdn* = 5) advertisement (*Z* = 5.54, *p* < 0.001), corresponding to a large effect size (partial eta^2^ = 0.29). There was large variation in the craving response among patients, as indicated by a relatively high standard deviation (SD = 18.30). However, in absolute terms, craving after alcohol-advertisement exposure was relatively low (see Fig. 2). Further, 38.8 % of patients did not show a higher craving level after alcohol-advertisement exposure as compared to craving after soda-advertisement exposure. Finally, a chi-square test to test whether the order in which the advertisement blocks were presented (alcohol first versus soft drink first) influenced whether participants showed a craving response or not did not reveal an order effect *X*^2^(1, *N* = 79) = 0.18, NS.

**Fig. 3:**
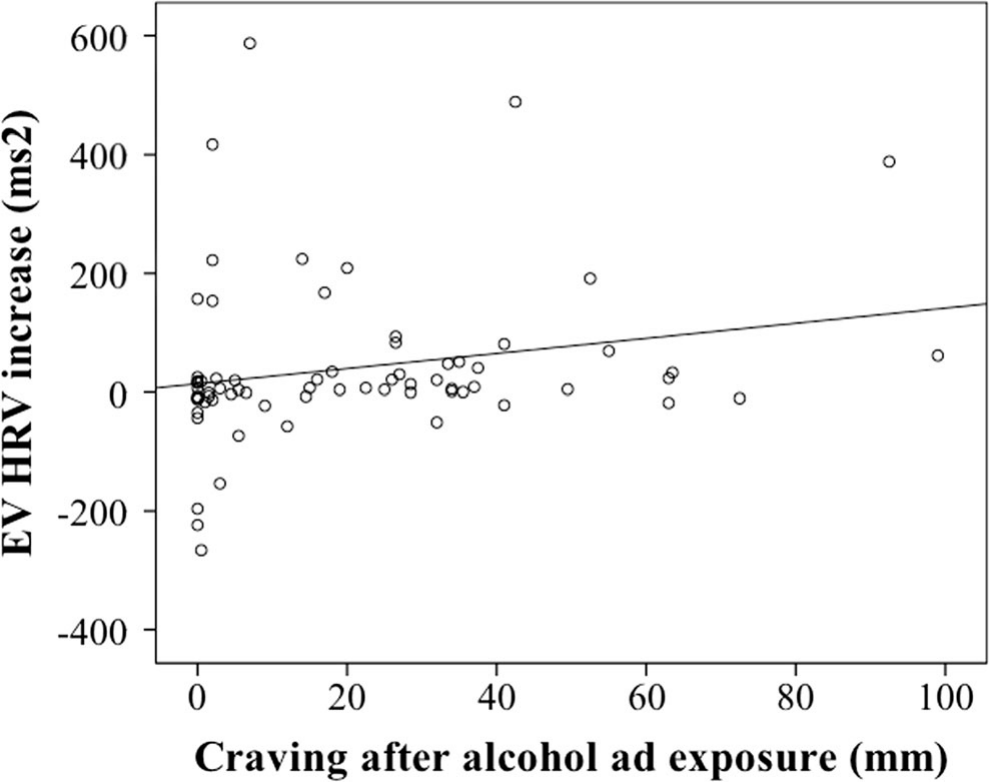
Scatterplot of the increase in EVHRV HF power during alcohol-cue exposure (as compared to pre-cue baseline) in alcohol advertisement (*y*-axis) against the craving scores after exposure to alcohol advertisement (*x*-axis)

**Fig. 4:**
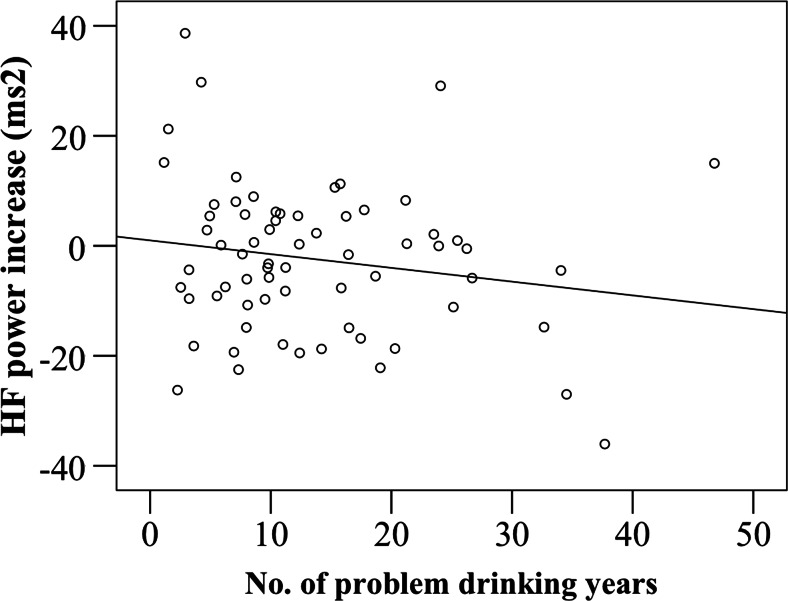
Scatterplot of the increase in EVHRV HF power during alcohol-cue exposure in alcohol advertisement (*y*-axis) against the number of problem-drinking years (*x*-axis)

Exploratory Spearman correlations indicated no significant correlation between the difference in craving after alcohol ads as compared to soft drink ads (i.e., relative craving) and the difference in mean HF HRV between the two blocks of advertisement (*r* = −0.04, *N* = 67, NS). Relative craving did not correlate significantly with the number of DSM-IV alcohol-dependence symptoms reported (*r* = 0.173, *N* = 76, NS) but absolute craving after alcohol-advertisement exposure showed a significant positive relationship (*r* = 0.252, *N* = 76, *p* = 0.028), indicating that higher craving levels were associated with more severe alcohol dependence. Furthermore, the increase in absolute EVHRV HF power during the presentation of alcohol cues (as compared to pre-cue baseline) showed a significant positive correlation with absolute craving after alcohol-advertisement exposure (*r* = 0.33, *N* = 71, *p* = 0.004), as can be seen in Fig. 3. Additionally, duration of problem drinking (number of problem-drinking years) correlated with increase in EVHRV HF power during the presentation of alcohol cues (*r* = −.26, *N* = 71, *p* = 0.03). Because age significantly correlated with the number of problem-drinking years, we performed a multiple linear regression analysis with age and number of problem-drinking years as predictors and EVHRV HF power cue reactivity as a dependent variable, revealing that only number of problem-drinking years was significantly negatively associated with cue reactivity. As can be seen in Fig. 4, the increase in EVHRV HF power was larger for patients with *shorter* histories of problematic drinking. Severity of alcohol dependence in the previous year (AUDIT) did not correlate with EVHRV HF power during presentation of alcohol cues.

### Field exposure to alcohol and soft drink advertisement

**Fig. 5:**
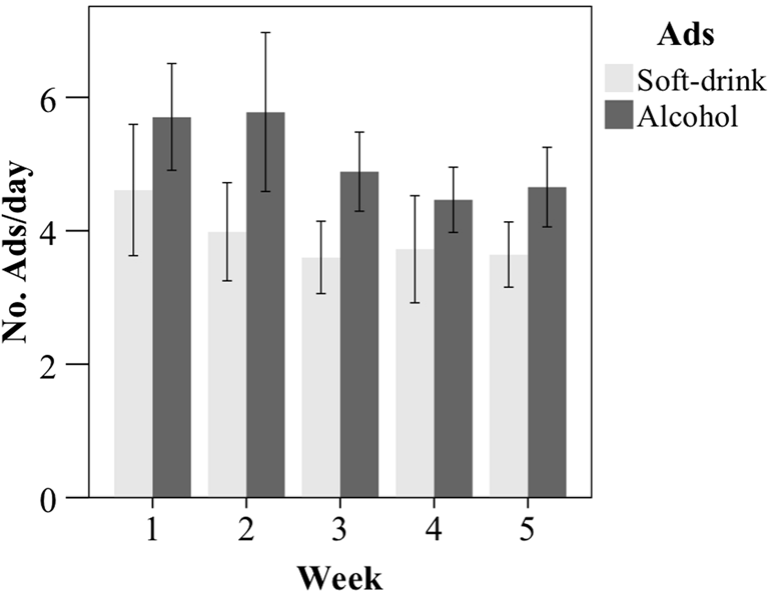
Mean number of self-reported exposures to soft-drink and alcohol advertisements per day for each of the five monitoring weeks. *Error bars* indicate the 95 % confidence interval

Mean exposure to alcohol and soft drink advertisement as reported in the diary for each of the 5 weeks in the interval between discharge and the first follow-up can be found in Fig. 5. Week-to-week reliability of total alcohol-advertisement exposure was high (Pearson correlations ranging from 0.65 to 0.87). Mean number of advertisement exposures was 5.38 per day for alcohol (SD = 4.14) and 4.05 per day for soft drink (SD = 4.14) over the whole 5-week monitoring period.

### Follow-up

Of the 68 patients who were enrolled in the longitudinal part of the study, 91 % could be retained at 5-week follow-up and 74 % at 3 months follow-up. Of the 58 patients who took part in the diary monitoring however, only 37 (63.8 %) returned the diary at the end of the monitoring period.

At 5 weeks and 3 months post-discharge, non-abstinence rates were 47.1 and 66.2 %, respectively. For binge drinking, relapse rates at 5 weeks and 3 months post-discharge were 25 and 39.7 %, respectively. Mean time to relapse was 27.12 (SD = 22.65) and 30.11 (SD = 23.19) days for non-abstinence and binge drinking, respectively. However, the mode was 21 and 14 days respectively, showing that a substantial number of patients relapsed within the first 3 weeks after discharge. Lastly, at 5 weeks and 3 months post-discharge, 29 and 42.9 % of patients, respectively, evaluated their current drinking behaviour as problematic.

Chi-square tests indicated that patients who did not return the diary had a significantly higher non-abstinence rate at 5 weeks (*X*^2^(1, *N* = 54) = 5.17, *p* = 0.02) and 3 months *X*^2^(1, *N* = 53) = 4.14, *p* = 0.04) follow-up than patients that returned the diary. Further, independent *t*-tests revealed that patients who did not return the diary had a significantly higher number of drinking days at 5 weeks (*T*(51) = 2.57, *p* = 0.02) and 3 months (*T*(52) = 2.25, *p* = 0.04) follow-up.

Because the baseline test only revealed a significant increase in EVHRV HF spectral power for alcohol cues against baseline, only this delta score was used as a cue reactivity predictor in the regression analyses. The omnibus-tests for the multiple regression analyses revealed only a significant amount of variance explained when the second block of predictors (EVHRV HF power increase for alcohol cues against baseline; difference in craving after the alcohol-advertisement block as compared to craving after the soda-advertisement block at baseline) was added for total number of drinks consumed as dependent (*F*(4,15) = 3.079, *p* = 0.049). However, the betas of the predictors failed to reach significance, with EVHRV HF power reaching the border of significance (*β* = −0.48, *p* = 0.052). Furthermore, for the imputed data-set, this result was non-significant. For all the other analyses, none of the predictors for neither the 5-week nor the 3-month follow-up predicted relapse variables ([*R*^2^] across both time points for each dependent in the imputed data-set; abstinence [0.08], binge-drinking status [0.13], number of binge-drinking days [0.055], time to first drink [*χ*^2^ (5) = 2.65).

Similarly for the interaction analyses, both for the original and imputed data, none of the analyses revealed a significant interaction between measures of baseline responsiveness to alcohol advertisement (i.e., EVHRV HF power for alcohol cues and the difference in craving after alcohol versus soft drink advertisement) and self-reported exposure to alcohol advertisement (i.e., the alcohol diary).

## Discussion

The present study investigated the nature of psychophysiological cue reactivity and craving in response to alcohol cues in alcohol dependence and its relation to subsequent drinking behaviour using a combined laboratory and field approach.

While patients were still in treatment, no significant mean HF HRV response to alcohol advertisements was observed as compared to a block of soft drink advertisements. Future studies could benefit from including a resting baseline condition that could then be subtracted from active exposure conditions, which might render an overall effect across conditions detectable. However, craving after alcohol-advertisement exposure was robustly elevated as compared to craving after soft drink advertisement exposure. It should be noted however that the median difference was only 9 mm (however, there was profound individual variation in the craving response) and that craving after an alcohol advertisement was still on the low end of the visual analogue scale. Therefore, the clinical applicability of this effect could be questioned. On the other hand, the absolute level of craving might have been blunted by the lack of availability of alcohol in the clinic relative to a more natural situation in which alcohol would be available (such as in the natural environment of patients). Furthermore, although craving at baseline did not predict relapse, absolute craving after alcohol-advertisement exposure did show a positive association with the number of alcohol-dependence symptoms, suggesting that cue-elicited craving may play a role in the development of alcohol dependence. Future studies could benefit from measuring craving pre- and post-exposure to alcohol advertisement and a control condition to verify that differences in craving between the blocks are not driven by a reduction in craving in the control condition.

A significant *increase* in EVHRV HF spectral power in response to the presentation of drug cues (as compared to pre-cue baseline) was found during alcohol advertisement in the present investigation. An increase in HF HRV power in response to drug cues has been found previously (Erblich et al. 2011; Garland 2011; but see Culbertson et al. 2010). There has been a debate on whether the increase in vagal tone represents an appetitive response (Garland 2011) or a regulatory homeostatic response to an aversive stimulus (Erblich et al. 2011) or both (Niaura et al. 1988; Wiers et al. 2007). Interestingly, a recent meta-analysis of studies examining cue reactivity to stress found a *decrease* in HF HRV in response to stress (Brindle et al. 2014). Further, a recent study also found an *increase* in HF HRV spectral power when confronting an obese population with high-caloric food (Udo et al. 2014). Therefore, HF HRV cue reactivity may represent a conditioned appetitive response to conditioned drug cues as has been proposed for cue reactivity as measured with fMRI (Tapert et al. 2003; Kühn 2011; Schacht et al. 2013). Additionally, the present HR data are in line with studies that have shown significant cue reactivity in response to drug cues as presented in tobacco (Vollstädt-Klein et al. 2011) and alcohol (George et al. 2001; Tapert et al. 2003) advertisements among nicotine- and alcohol-dependent patients, respectively. Further, our results are in keeping with the suggestion that the presentation of drug cues and scenes depicting (preparation of) drug use may be driving such conditioned physiological cue reactivity (Mucha et al. 2000).

A significant but moderate association was observed between the EVHRV HF power increase during alcohol-cue exposure and absolute craving after alcohol-advertisement exposure. Such modest associations between physiological cue reactivity and craving have been observed previously (Myrick et al. 2003; Wrase et al. 2007; Mason et al. 2008). Therefore, craving and physiological cue reactivity may represent partially overlapping phenomena. It has been suggested that cue reactivity is a primary response of the nervous system to conditioned drug cues, as predicted by incentive salience theory (Robinson and Berridge 2008) and that craving represents additional processes such as the interpretation of this response or the will to resist drinking (Rohsenow et al. 1994; Drummond 2000) which may be influenced by various organismic and contextual factors, resulting in an association of modest magnitude between the two phenomena. Together, the baseline measures suggest that alcohol advertisement has generic (non brand-specific) cue reactivity and craving effects in alcohol-dependent patients. The results furthermore suggest that physiological cue reactivity and craving effects of alcohol advertisement are driven by portrayal of drug cues such as presentation of the drug (i.e., alcohol), individuals preparing to drink and actual drinking behaviour. An interesting avenue for future studies could be to measure craving throughout the block of alcohol advertisements to observe how craving is affected by alcohol cues in time, and how craving dynamics corresponds to HF HRV dynamics.

During the 5-week follow-up period after discharge, patients reported being exposed to a mean of five alcohol advertisements per day. Week-to-week reliability of the diary was high, suggesting that a diary may be a sensitive method to assess exposure to an alcohol advertisement in the field. However, there was a substantial drop-out in the diary measure. Therefore, future investigations could use an electronic version of the diary (allowing for instant data collection) and could reduce the number of monitoring days to increase data retention and reduce drop-out rates. Also, an important step forward would be to test the validity of the diary. Taking the baseline laboratory and follow-up field results together then, although tentative, our results suggest that alcohol-dependent patients may experience cue reactivity and craving as a result of alcohol-advertisement exposure on a daily basis.

Even when using a relatively conservative definition of relapse, i.e., the occurrence of at least one binge-drinking episode, relapse rates were high in the present study, with two thirds of patients reporting non-abstinence at the 3-month follow-up, in line with previous work (Witkiewitz and Marlatt 2007). However, baseline physiological cue reactivity and cue-elicited craving did not predict relapse in the present study. Regarding HF HRV cue reactivity, one previous study did find a relationship with relapse, with patients showing a greater increase in HF spectral power of HRV after alcohol cue exposure having a higher probability of relapse (Garland et al. 2012). However, in this study patients were first exposed to a stressor before being exposed to alcohol cues. Thus, it might specifically be the HF HRV response to stress-primed alcohol cues that is predictive of relapse. Regarding the relationship between baseline cue-elicited craving and subsequent relapse, previous results have been mixed, with three studies finding a positive relationship (Cooney et al. 1997; Seo et al. 2013; Papachristou et al. 2014) but one study failing to do so (Rohsenow et al. 1994). Interestingly, the magnitude of alcohol craving in response to stress might be particularly predictive of future drinking as well, as two of the three studies above that did find a relationship used a (negative) mood induction procedure before measuring alcohol craving (Cooney et al. 1997; Seo et al. 2013). A final explanation that has been suggested previously is that in severe drug dependence (as was the case in the present study), external cues play a less important role in determining craving and drug use, as behaviour in this advanced stage of dependence is governed by internal (withdrawal) cues (Vollstädt-Klein et al. 2011) or habit (Everitt and Robbins 2005) rather than an appetitive response to external drug cues. In (indirect) support of this interpretation, we found a reduced EVHRV HF power response to alcohol cues with longer problematic drinking histories in the present investigation.

In addition to the *magnitude* of the cue reactivity and the craving response to drug cues at baseline, we were interested in whether the *degree of actual exposure* to alcohol cues in the natural environment may show a relationship with relapse. Although patients reported a substantial daily exposure to alcohol advertisement, no robust relationship between the degree of exposure to alcohol advertisement and drinking behaviour was found. However, several factors could have obscured an existing relationship between alcohol-advertisement exposure and drinking behaviour. First, there was a relatively large and selective drop-out from the diary monitoring part of the study, which may have reduced power to detect the relationship and may have distorted the observed relationship. Second, society is saturated with alcohol advertisement, resulting in low variation in the dose of advertisement among individuals and therefore reducing power to detect the relationship through restriction of range. Alternatively, as suggested above, external cues may play a minor role in severe alcohol dependence (Vollstädt-Klein et al. 2011). All in all then, concerning the relationship between alcohol advertisement and relapse, our results should be taken as preliminary.

### Conclusions

Alcohol-cue exposure, and more specifically alcohol advertisement, causes a robust craving response in alcohol-dependent patients. Further, display of the drug (i.e., an alcoholic beverage), individuals preparing to drink and actual drinking behaviour seem to drive physiological cue reactivity and craving in response to alcohol advertisement, likely through an appetitive conditioned response, as predicted by incentive salience theory. A practical implication of our results is that reducing alcohol cues in advertisement could therefore theoretically reduce the occurrence of episodes of acute craving and cue reactivity in alcohol-dependent patients.

## References

[CR1] Bechara A (2005). Decision making, impulse control and loss of willpower to resist drugs: a neurocognitive perspective. Nat Neurosci.

[CR2] Beck A, Wüstenberg T, Genauck A, Schlagenhauf F, Smolka MN, Mann K, Heinz A (2012). Effect of brain structure, brain function, and brain connectivity on relapse in alcohol-dependent patients. Arch Gen Psychiatry.

[CR3] Brindle RC, Ginty AT, Phillips AC, Carroll D (2014). A tale of two mechanisms: a meta-analytic approach toward understanding the autonomic basis of cardiovascular reactivity to acute psychological stress. Psychophysiology.

[CR4] Carter BL, Tiffany ST (1999). Meta-analysis of cue-reactivity in addiction research. Addiction.

[CR5] Collins LM, Schafer JL, Kam CM (2001). A comparison of inclusive and restrictive strategies in modern missing data procedures. Psychol Methods.

[CR6] Cooney NL, Litt MD, Morse PA, Bauer LO, Gaupp L (1997). Alcohol cue reactivity, negative-mood reactivity, and relapse in treated alcoholic men. J Abnorm Psychol.

[CR7] Culbertson C, Nicolas S, Zaharovits I, London ED, De La Garza R, Brody AL, Newton TF (2010). Methamphetamine craving induced in an online virtual reality environment. Pharmacol Biochem Behav.

[CR8] Drummond DC (2000). What does cue-reactivity have to offer clinical research?. Addiction.

[CR9] Erblich J, Bovbjerg DH, Sloan RP (2011). Exposure to smoking cues: cardiovascular and autonomic effects. Addict Behav.

[CR10] Bernston GG, Bigger JT, Eckberg D (1997). Heart rate variability: origins, methods, and interprative caveats. Psyhcophysiology.

[CR11] Everitt BJ, Robbins TW (2005). Neural systems of reinforcement for drug addiction: from actions to habits to compulsion. Nat Neurosci.

[CR12] Garland EL (2011). Trait mindfulness predicts attentional and autonomic regulation of alcohol cue-reactivity. J Psychophysiol.

[CR13] Garland EL, Franken I, Howard M (2012). Cue-elicited heart rate variability and attentional bias predict alcohol relapse following treatment. Psychopharmacology.

[CR14] George MS, Anton RF, Bloomer C, Teneback C, Drobes DJ, Lorberbaum JP, Nahas Z, Vincent DJ (2001). Activation of prefrontal cortex and anterior thalamus in alcoholic subjects on exposure to alcohol-specific cues. Arch Gen Psychiatry.

[CR15] Grüsser SM, Wrase J, Klein S, Hermann D, Smolka MN, Ruf M, Weber-Fahr W, Flor H, Braus DF, Heinz A (2004). Cue-induced activation of the striatum and medial prefrontal cortex is associated with subsequent relapse in abstinent alcoholics. Psychopharmacology.

[CR16] Hallgren KA, Witkiewitz K (2013). Missing data in alcohol clinical trials: a comparison of methods. Alcohol Clin Exp Res.

[CR17] Heinz A, Wrase J, Kahnt T, Beck A, Bromand Z, Grüsser SM, Kienast T, Smolka MN, Flor H, Mann K (2007) Brain activation elicited by affectively positive stimuli is associated with a lower risk of relapse in detoxified alcoholic subjects. Alcohol: Clin Exp Res 31:1138–1147 10.1111/j.1530-0277.2007.00406.x 17488322

[CR18] Joos L, Schmaal L, Goudriaan AE, Fransen E, Van den Brink W, Sabbe BGC, Dom G (2012). Age of onset and neuropsychological functioning in alcohol dependent inpatients. Alcohol Clin Exp Res.

[CR19] Koob GF, Volkow ND (2009). Neurocircuitry of addiction. Neuropsychopharmacology.

[CR20] Kozlowski LT, Pillitteri JL, Sweeney CT, Whitfield KE, Graham JW (1996). Asking questions about urges or cravings for cigarettes. Psychol Addict Behav.

[CR21] Kühn SJ (2011). Common biology of craving across legal and illegal drugs – a quantitative meta-analysis of cue-reactivity brain response. Eur J Neurosci.

[CR22] Lit MD, Cooney NL (1999). Inducing craving for alcohol in the laboratory. Alcohol Res Health.

[CR23] Llabre MM, Spitzer SB, Saab PG, Ironson GH, Schneiderman N (1991). The reliability and specificity of delta versus residualized change as measures of cardiovascular reactivity to behavioral challenges. Psychophysiology.

[CR24] Mason B, Light J, Escher T, Drobes D (2008). Effect of positive and negative affective stimuli and beverage cues on measures of craving in non treatment-seeking alcoholics. Psychopharmacology.

[CR25] May J, Kavanagh DJ, Andrade J (2015). The elaborated intrusion theory of desire: a 10-year retrospective and implications for addiction treatments. Addict Behav.

[CR26] Mucha RF, Geier A, Stuhlinger M, Mundle G (2000). Appetitive effects of drug cues modelled by pictures of the intake ritual: generality of cue-modulated startle examined with inpatient alcoholics. Psychopharmacology.

[CR27] Myrick H, Anton RF, Li X, Henderson S, Drobes D, Voronin K, George MS (2003). Differential brain activity in alcoholics and social drinkers to alcohol cues: relationship to craving. Neuropsychopharmacology.

[CR28] Niaura RS, Rohsenow DJ, Binkoff JA, Monti PM, Pedraza M, Abrams DB (1988). Relevance of cue reactivity to understanding alcohol and smoking relapse. J Abnorm Psychol.

[CR29] Papachristou H, Nederkoorn C, Havermans R, Bongers P, Beunen S, Jansen A (2013). Higher levels of trait impulsiveness and a less effective response inhibition are linked to more intense cue-elicited craving for alcohol in alcohol-dependent patients. Psychopharmacology.

[CR30] Papachristou H, Nederkoorn C, Giesen JCAH, Jansen A (2014). Cue reactivity during treatment, and not impulsivity, predicts an initial lapse after treatment in alcohol use disorders. Addict Behav.

[CR31] Patrick ME, Lee CM (2010). Comparing numbers of drinks: college students' reports from retrospective summary, followback, and prospective daily diary measures. J Stud Alcohol Drugs.

[CR32] Quintana DS, McGregor IS, Guastella AJ, Malhi GS, Kemp AH (2013) A meta-analysis on the impact of alcohol dependence on short-term resting-state heart rate variability: implications for cardiovascular risk. Alcohol: Clin Exp Res 10.1111/j.1530-0277.2012.01913.x 22834996

[CR33] Robinson TE, Berridge KC (2008). The incentive sensitization theory of addiction: some current issues. Philos Trans Royal Soc B: Biol Sci.

[CR34] Rohsenow DJ, Monti PM, Rubonis AV, Sirota AD, Niaura RS, Colby SM, Wunschel SM, Abrams DB (1994). Cue reactivity as a predictor of drinking among male alcoholics. J Consult Clin Psychol.

[CR35] Saunders JB, Aasland OG, Babor TF, De La Fuente JR, Grant M (1993). Development of the alcohol use disorders identification test (audit): who collaborative project on early detection of persons with harmful alcohol consumption-ii. Addiction.

[CR36] Schacht JP, Anton RF, Myrick H (2013). Functional neuroimaging studies of alcohol cue reactivity: a quantitative meta-analysis and systematic review. Addict Biol.

[CR37] Seo DL, Lacadie CM, Tuit K, Hong KL, Constable RT, Sinha R (2013). Disrupted ventromedial prefrontal function, alcohol craving, and subsequent relapse risk. JAMA Psychiatry.

[CR38] Sheehan DV, Lecrubier Y, Sheehan KH (1998). The Mini-International Neuropsychiatric Interview (M I N I): the development and validation of a structured diagnostic psychiatric interview for DSM-IV and ICD-10. J Clin Psychiatry.

[CR39] Slater M, Antley A, Davison A, Swapp D, Guger C, Barker C, Pistrang N, Sanchez-Vives MV (2006). A virtual reprise of the Stanley Milgram obedience experiments. PLoS One.

[CR40] Sobell LC, Agrawal S, Sobell MB, Leo GI, Young LJ, Cunningham JA, Simco ER (2003). Comparison of a quick drinking screen with the timeline followback for individuals with alcohol problems. J Stud Alcohol.

[CR41] Staiger PK, White JM (1991). Cue reactivity in alcohol abusers: stimulus specificity and extinction of the responses. Addict Behav.

[CR42] Tapert SF, Cheung EH, Brown GG, Frank LR, Paulus MP, Schweinsburg AD, Meloy MJ, Brown SA (2003). Neural response to alcohol stimuli in adolescents with alcohol use disorder. Arch Gen Psychiatry.

[CR43] Thayer JF, Lane RD (2000). A model of neurovisceral integration in emotion regulation and dysregulation. J Affect Disord.

[CR44] Thayer JF, Soller JJ, Ruiz-Padial E, Vila J (2002). Estimating respiratory frequency from autoregressive spectral analysis of heart period. IEEE Eng Med Biol.

[CR45] Udo T, Weinberger AH, Grilo CM, Brownell KD, DiLeone RJ, Lampert R, Matlin SL, Yanagisawa K, McKee SA (2014). Heightened vagal activity during high-calorie food presentation in obese compared with non-obese individuals—results of a pilot study. Obes Res Clin Pract.

[CR46] van Vliet IM, de Beurs E (2007). The MINI-International Neuropsychiatric Interview. A brief structured diagnostic psychiatric interview for DSM-IV en ICD-10 psychiatric disorders. Tijdschrift voor de Psychiatrie [J Psychiatr].

[CR47] Verdejo-Garcia A, Clark L, Dunn BD (2012). The role of interoception in addiction: a critical review. Neurosci Biobehav Rev.

[CR48] Volkow ND, Fowler JS, Wang GJ, Telang F, Logan J, Jayne M, Ma Y, Pradhan K, Swanson JM (2010). Cognitive control of drug craving inhibits brain reward regions in cocaine abusers. Neuroimage.

[CR49] Volkow ND, Wang G-J, Fowler JS, Tomasi D (2012). Addiction circuitry in the human brain. Annu Rev Pharmacol Toxicol.

[CR50] Vollstädt-Klein S, Kobiella A, Bühler M, Graf C, Fehr C, Mann, K, Smolka MN (2011) Severity of dependence modulates smokers’ neuronal cue reactivity and cigarette craving elicited by tobacco advertisement. Addict Biol 16:166–175 10.1111/j.1369-1600.2010.00207.x 20331560

[CR51] Wallén M, Hasson D, Theorell T, Canlon B, Osika W (2012) Possibilities and limitations of the polar RS800 in measuring heart rate variability at rest. Eur J Appl Physiol Occup Physiol 112:1153–1165 10.1007/s00421-011-2079-9 21766225

[CR52] Weippert M, Kumar M, Kreuzfeld S, Arndt D, Rieger A, Stoll R (2010) Comparison of three mobile devices for measuring R–R intervals and heart rate variability: Polar S810i, Suunto t6 and an ambulatory ECG system. Eur J Appl Physiol 109:779–786 10.1007/s00421-010-1415-9 20225081

[CR53] Wiers RW, Bartholow BD, van den Wildenberg E, Thush C, Engels RC, Sher KJ, Grenard J, Ames SL, Stacy AW (2007) Automatic and controlled processes and the development of addictive behaviors in adolescents: a review and a model. Pharmacol Biochem Behav 86:263–283 10.1016/j.pbb.2006.09.021 17116324

[CR54] Witkiewitz K, Marlatt GA (2007) Modeling the complexity of post-treatment drinking: It’s a rocky road to relapse. Clin Psychol Rev 27:724–738 10.1016/j.cpr.2007.01.002 PMC1995671 17355897

[CR55] Wrase J, Schlagenhauf F, Kienast T, Wüstenberg T, Bermpohl F, Kahnt T, Beck A, Ströhle A, Juckel G, Knutson B, Heinz A (2007) Dysfunction of reward processing correlates with alcohol craving in detoxified alcoholics. NeuroImage 35:787–794 10.1016/j.neuroimage.2006.11.043 17291784

